# Combined Bilateral Salpingo-oophorectomy and Cesarean Delivery in *BRCA1/2* Alteration Carriers: A Case Series

**DOI:** 10.1097/AOG.0000000000005423

**Published:** 2023-11-02

**Authors:** Victoria E. Barker, Evangelia Vlachodimitropoulou, Patrick O’Brien, Joseph Iskaros, Adam N. Rosenthal

**Affiliations:** University College London Hospitals, National Health Service Foundation Trust, and the Department of Women’s Cancer, University College London Elizabeth Garrett Anderson Institute for Women's Health, University College London, London, United Kingdom.

## Abstract

**BACKGROUND:**

The cumulative lifetime risk of ovarian cancer is 16–68% and 11–30% in female *BRCA1* and *BRCA2* gene alteration carriers, respectively. Risk-reducing bilateral salpingo-oophorectomy (RRSO) is the only proven way to reduce ovarian cancer mortality. We report a series of patients who underwent risk-reducing surgery at the time of planned obstetric-indicated cesarean delivery.

**CASES:**

This is a case series of four women carrying a pathogenic germline *BRCA1* or *BRCA2* gene alteration who underwent RRSO at the time of cesarean delivery between March 1, 2018, and March 31, 2022. All women were referred during pregnancy to the University College London Hospitals Familial Cancer Clinic for consideration of RRSO at the time of obstetric-indicated cesarean delivery. Women were considered eligible for RRSO if they had a proven pathogenic germline alteration, would have completed childbearing after the cesarean delivery, and were older than age 35 or 40 years with *BRCA1* or *BRCA2* alterations, respectively. Operating time, blood loss, transfusion requirements, length of hospital stay, complications, and ability to breastfeed were assessed and, where possible, compared with the institutional means for similar patients who underwent cesarean delivery only, to determine whether RRSO was associated with increased morbidity. Women were contacted 11–59 months postprocedure to assess satisfaction. The mean blood loss was 687 mL (range 400–1,000 mL), mean operating time was 68 minutes, mean length of hospital stay was 3 days, and mean change in hemoglobin was −1 g/dL. No patient required a transfusion, had internal organ damage, returned to the operating room, or was readmitted. One of two women with intact breast tissue successfully breastfed, and the other chose to bottle feed. The mean contemporaneous institutional blood loss for cesarean delivery was not significantly different at 681 mL for singleton pregnancies and 872 mL for twin pregnancies. All four women reported a high level of satisfaction with the combined procedure.

**CONCLUSION:**

Our results show that RRSO can be performed at the time of cesarean delivery with high patient satisfaction. This approach can be offered to appropriately counseled individuals, with the benefit of avoiding the need for two separate procedures, with potentially reduced patient morbidity and health care costs.

## Abstract 2

Risk-reducing salpingo-oophorectomy at the time of planned obstetric-indicated cesarean delivery can be offered to appropriately counseled *BRCA1/2* alteration carriers.

**Teaching Points**

1. Risk-reducing bilateral salpingo-oophorectomy can be offered to appropriately selected and counseled individuals with *BRCA1/2* gene alterations at the time of a planned obstetric-indicated cesarean delivery.
2. The pathologist should be made aware that the patient is a *BRCA* carrier, and the SEE-FIM protocol (Sectioning and Extensively Examining the FIMbriated end) should be followed to minimize the chance of missing an occult tubal or ovarian cancer.
3. Transdermal continuous combined hormone therapy should be started 6 weeks postnatally (or later in those able and desiring to breastfeed) and continued to the age of 51 years unless there are contraindications.

Ovarian cancer is the most lethal gynecologic malignancy and continues to have a poor prognosis, largely attributable to late-stage at presentation.^1^ *BRCA* gene alterations are associated with approximately 15% of ovarian cancers.^2^ The cumulative ovarian cancer lifetime risk is 16–68% and 11–30% for *BRCA1* and *BRCA2* alteration carriers, respectively.^3^ Identifying individuals with a genetic predisposition and performing risk-reducing bilateral salpingo-oophorectomy (RRSO) offers a significant opportunity for ovarian cancer prevention and mortality reduction.^4^ We report a series of women who underwent RRSO at the time of planned obstetric-indicated cesarean delivery.

## CASES

This is a case series of four patients with *BRCA* gene alterations, referred during pregnancy to the University College London Hospitals Familial Cancer Clinic for consideration of RRSO at the time of planned obstetric-indicated cesarean delivery between March 1, 2018, and March 31, 2022. All four were receiving maternity care at the same institution. After their consultations, each individual’s case was discussed at a multidisciplinary familial cancer meeting, including gynecologic oncologists, clinical geneticists, genetic counselors, and psychologists. Individuals were considered eligible for such surgery if they had a proven pathogenic germline gene alteration, would have completed childbearing after cesarean delivery, and were older than age 35 or 40 years with *BRCA1* or *BRCA2* alterations, respectively. A note was put onto the electronic health care record with information for the emergency obstetric team in the event the patient required emergency cesarean delivery before the planned cesarean delivery date. Operative time, blood loss, change in hemoglobin concentration, perioperative complications, length of hospital stay, and ability to breastfeed were assessed. Patients were contacted by telephone postprocedure, and responses were recorded. Satisfaction was measured by asking each patient, “Overall how happy were you having risk-reducing surgery at the time of cesarean?”; the exact words used by the patient were recorded.

Three patients had an alteration in *BRCA1* and one in *BRCA2* (Table 1). The mean age was 42.5 years (range 40–45 years). Three patients had had breast cancer, and three conceived with in vitro fertilization. Two patients were primigravid and had dichorionic diamniotic twin pregnancies (both in vitro fertilization), and two were multiparous; each had one previous cesarean delivery. The mean gestational age at delivery was 38 1/7 weeks (range 37 4/7–39 0/7 weeks). The obstetric indications for cesarean delivery were multiple pregnancy (n=2) and previous cesarean delivery (n=2).

**Table 1.**
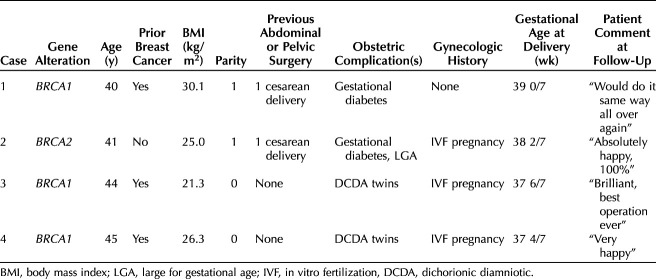
Patient Characteristics

All surgeries were performed under spinal anesthesia. The mean estimated blood loss was 687 mL (range 400–1,000 mL), compared with a mean contemporaneous institutional blood loss for cesarean delivery of 681 mL and 872 mL for singleton and twin pregnancies, respectively. Blood loss was calculated by weighing swabs and measuring the volume in the suction. The mean change in hemoglobin was −1 g/dL. The mean operating time was 68 minutes (range 46–90 minutes). Histology of the surgical specimens, analyzed according to the SEE-FIM protocol (Sectioning and Extensively Examining the FIMbriated end), confirmed complete removal of the fallopian tubes and ovaries, with no evidence of dysplasia or malignancy. The mean length of hospital stay was 3 days (range 2–4 days). No patient required blood transfusion, had internal organ damage, required reoperation, or was readmitted. Two patients had undergone bilateral mastectomy for breast cancer. The third patient with prior breast cancer, treated with lumpectomy, chemotherapy, and radiotherapy, successfully breastfed from her nonirradiated breast for 1 month. The patient with no history of breast cancer chose to bottle feed. Three of the four patients used hormone therapy (HT) postnatally; two with prior triple-negative breast cancer and one with no prior breast cancer.

Two patients had minor postnatal problems: one experienced detachment of transdermal HT patches and switched to estradiol gel and an oral progestogen. Another patient had three episodes of vaginal bleeding 4–7 months postdelivery despite no HT being prescribed due to previous breast cancer. No cause for the bleeding was found on examination or imaging. Her follicle-stimulating hormone level was 47.6 international units/mL, and she had menopausal symptoms. The bleeding resolved without intervention. All four women reported being very satisfied with the combined surgery 11–59 months postprocedure (Table 1).

## DISCUSSION

Patients with inherited *BRCA* gene alterations have an increased risk of ovarian cancer, exceeding 5% in the early 40s for *BRCA1* carriers and in the early 50s for *BRCA2* carriers. General population ovarian cancer screening does not reduce mortality from the disease.^5^ Therefore, women at high risk are recommended to undergo RRSO,^6^ which reduces ovarian cancer risk by at least 90%. Risk-reducing bilateral salpingo-oophorectomy should be offered from age 35 years and 40 years in *BRCA1* and *BRCA2* carriers, respectively.^7^ If there are very early-onset ovarian cancers (younger than age 40 years in *BRCA1* carriers and younger than age 45 years in *BRCA2* carriers) in the individual's family, these ages may be reduced pragmatically to 5 years younger than the age of onset of the earliest ovarian cancer (but not breast cancer) in the family.

Worldwide, 21.1% of women give birth by cesarean delivery.^8^ This rate has increased and is expected to continue to rise for a variety of reasons, including maternal request, prior cesarean delivery, obstetric culture, increased maternal age, increased prepregnancy morbidity, and increased perceived risk of vaginal birth. We believe that the option of combined RRSO and cesarean delivery should be offered to all pregnant *BRCA* carriers requiring cesarean delivery who have completed childbearing and are at an appropriate age for RRSO, after discussion of the advantages and disadvantages.

Risk-reducing bilateral salpingo-oophorectomy has been shown to be cost effective for *BRCA1* and *BRCA2* carriers.^9^ Performing this procedure at the time of planned obstetric-indicated cesarean delivery potentially could provide additional cost benefits given the avoidance of a separate operation, with its attendant expense.

It is important that individuals are counseled thoroughly and that each case is discussed, ideally at a multidisciplinary meeting involving obstetricians, gynecologic oncologists, clinical geneticists, genetic counselors, and clinical psychologists. Important considerations include the theoretical (but unconfirmed) possibility of additional blood loss at cesarean delivery due to engorgement of pelvic vessels in pregnancy, infertility, the effects of iatrogenic menopause, and the need for HT in individuals without prior breast cancer (Appendix 1, available online at http://links.lww.com/AOG/D454).

Symptoms of surgical menopause tend to be similar to those of natural menopause but can be more severe. The usual hypoestrogenic postpartum state means that individuals might experience menopausal symptoms regardless of whether RRSO was performed. It is unknown whether sudden loss of ovarian function worsens these symptoms in the puerperium. In addition, in the longer term, surgical menopause increases rates of coronary heart disease, cognitive impairment, stroke, sexual dysfunction, and osteoporosis.^10^ Current evidence supports the use of HT until age 51 years in individuals with *BRCA* alterations unless there are contraindications, such as prior breast cancer. Even then, further discussion with the patient's oncologist is recommended to examine the risks and benefits of HT; for example, those with triple-negative breast cancer or those in whom menopausal symptoms severely affect quality of life may be candidates for HT. If required, individuals can be referred to a menopause specialist.^11^

There is a lack of evidence on the optimal time to start HT postdelivery. It is known that there is a substantially increased risk of venous thromboembolism (VTE) (22 additional events/100,000 deliveries) in the *postpartum period*, which is traditionally defined as the 6 weeks after delivery. There is some evidence to suggest that elevated VTE risk persists at a lower level (3 additional events/100,000 deliveries) up to 12 weeks postpartum.^12^ Transdermal rather than oral HT started 6 weeks postdelivery (or later in those able and desiring to breastfeed) should be considered as the first-line option because of the reduced risk of VTE with transdermal HT. The effects of RRSO performed at cesarean delivery on breastfeeding have not been well-studied. However, breastfeeding is not thought to be dependent on ovarian sex steroids but rather on pituitary-derived oxytocin and prolactin. Hormone therapy suppresses lactation and can cause jaundice and breast enlargement in the neonate, so it should be withheld until the child is weaned.^13^

With growing evidence that the fallopian tubes are the origin of the majority of high-grade serous ovarian cancers,^14^ risk-reducing early salpingectomy and delayed oophorectomy has been suggested as a two-stage alternative to RRSO. The fallopian tubes are removed on completion of childbearing, and the ovaries are subsequently removed around the age of natural menopause (51 years). This has the advantage of offering some level of protection from ovarian cancer while potentially avoiding the sequelae of early menopause and the need for HT. Risk-reducing early salpingectomy and delayed oophorectomy is currently being offered in ongoing clinical trials.^15^ Until evidence regarding the safety of risk-reducing early salpingectomy and delayed oophorectomy is confirmed, it is recommended that it be offered only as part of a clinical trial. However, *BRCA* carriers requesting sterilization at the time of cesarean delivery should be offered full salpingectomy rather than partial bilateral salpingectomy or tubal ligation. Video 1 shows salpingectomy at the time of cesarean delivery.

In conclusion RRSO can be performed at the time of planned obstetric-indicated cesarean delivery. This approach can be offered to appropriately counseled individuals.
